# Untargeted Metabolomics and Body Mass in Adolescents: A Cross-Sectional and Longitudinal Analysis

**DOI:** 10.3390/metabo13080899

**Published:** 2023-07-30

**Authors:** Amarnath Singh, Garrett Kinnebrew, Ping-Ching Hsu, Daniel Y. Weng, Min-Ae Song, Sarah A. Reisinger, Joseph P. McElroy, Brittney Keller-Hamilton, Amy K. Ferketich, Jo L. Freudenheim, Peter G. Shields

**Affiliations:** 1 Comprehensive Cancer Center, The Ohio State University, Columbus, OH 43210-1240, USA; amarnath.singh@osumc.edu (A.S.); daniel.weng@osumc.edu (D.Y.W.); 2 Department of Biomedical Informatics, Biomedical Informatics Shared Resources (BISR), The Ohio State University, Columbus, OH 43210-1240, USA; garrett.kinnebrew@osumc.edu; 3 Department of Environmental Health Sciences, Fay W. Boozman College of Public Health, University of Arkansas for Medical Sciences, Little Rock, AR 72205, USA; phsu@uams.edu; 4 College of Public Health, The Ohio State University, Columbus, OH 43210-1240, USA; song.991@osu.edu (M.-A.S.); ferketich.1@osu.edu (A.K.F.); 5 Center for Tobacco Research, Comprehensive Cancer Center, The Ohio State University, Columbus, OH 43210-1240, USA; sarah.reisinger@osumc.edu (S.A.R.); brittney.keller-hamilton@osumc.edu (B.K.-H.); 6 Center for Biostatistics, Department of Biomedical Informatics, The Ohio State University, Columbus, OH 43210-1240, USA; joseph.mcelroy@osumc.edu; 7 Department of Internal Medicine, College of Medicine, The Ohio State University, Columbus, OH 43210-1240, USA; 8 Department of Epidemiology and Environmental Health, University at Buffalo, Buffalo, NY 14214, USA; jfreuden@buffalo.edu

**Keywords:** BMI z-score, obesity, metabolome, adolescent obesity, untargeted metabolomics

## Abstract

Obesity in children and adolescents has increased globally. Increased body mass index (BMI) during adolescence carries significant long-term adverse health outcomes, including chronic diseases such as cardiovascular disease, stroke, diabetes, and cancer. Little is known about the metabolic consequences of changes in BMI in adolescents outside of typical clinical parameters. Here, we used untargeted metabolomics to assess changing BMI in male adolescents. Untargeted metabolomic profiling was performed on urine samples from 360 adolescents using UPLC–QTOF-MS. The study includes a baseline of 235 subjects in a discovery set and 125 subjects in a validation set. Of them, a follow-up of 81 subjects (1 year later) as a replication set was studied. Linear regression analysis models were used to estimate the associations of metabolic features with BMI z-score in the discovery and validation sets, after adjusting for age, race, and total energy intake (kcal) at false-discovery-rate correction (FDR) ≤ 0.1. We identified 221 and 16 significant metabolic features in the discovery and in the validation set, respectively. The metabolites associated with BMI z-score in validation sets are glycylproline, citrulline, 4-vinylsyringol, 3′-sialyllactose, estrone sulfate, carnosine, formiminoglutamic acid, 4-hydroxyproline, hydroxyprolyl-asparagine, 2-hexenoylcarnitine, L-glutamine, inosine, N-(2-Hydroxyphenyl) acetamide glucuronide, and galactosylhydroxylysine. Of those 16 features, 9 significant metabolic features were associated with a positive change in BMI in the replication set 1 year later. Histidine and arginine metabolism were the most affected metabolic pathways. Our findings suggest that obesity and its metabolic outcomes in the urine metabolome of children are linked to altered amino acids, lipid, and carbohydrate metabolism. These identified metabolites may serve as biomarkers and aid in the investigation of obesity’s underlying pathological mechanisms. Whether these features are associated with the development of obesity, or a consequence of changing BMI, requires further study.

## 1. Introduction

More than 379 million children and adolescents worldwide are overweight or obese [1]. In the United States from 2017 to 2020, the prevalence of obesity was 19.7% in the age group of 2–19 years and affected about 14.7 million children and adolescents [2]. Up to 80% of obese children become obese adults, indicating that childhood obesity typically extends into adulthood [3]. Obesity is a common, serious, and multifactorial disease associated with genetics [4], environment [5], physiology [6], increased consumption of hypercaloric foods [7], and sedentary lifestyles [8]. Increased body mass index (BMI) and obesity during adolescence carry significant long-term adverse health outcomes, including the development of chronic diseases and mortality, such as cardiovascular disease, stroke, insulin resistance diabetes, and cancer [9,10,11,12,13]. Therefore, efficient interventions and predictive biomarkers for the development of obesity are needed. As children grow, the assessment of body mass index (BMI) requires adjustment for age and gender [14]. While a study of BMI typically categorizes adolescents as normal, overweight, or obese based on BMI percentile [15,16], a better measure of body mass index is the BMI z-score as a continuous variable because the BMI z-score is age and gender-specific [17]. In this study, we used the BMI z-score to investigate the association between the urinary metabolome and obesity.

Untargeted metabolomics profiles low-molecular-weight metabolites (<1500 Da) in biospecimens to elucidate cell physiology and disease mechanisms, and to identify biomarkers of disease risk [18,19]. This approach helps identify the molecular mechanisms of complex diseases, as well as for disease monitoring and risk assessment. In the past, targeted and untargeted metabolomics studies have been used to study the metabolome signature of obesity in response to dietary intake [20], the effects of specific dietary patterns or weight loss [21,22] or gain interventions, and body fat (%) [23]. 

In our comprehensive literature review, we identified 41 studies on adolescent metabolomics and obesity [10]. Out of the total studies, 33 specifically focused on blood (plasma, serum), 3 studies examined umbilical cord blood, and 1 study used saliva samples, while 6 studies utilized urine samples [10,24,25,26,27,28,29]. Urine contains diverse metabolites reflecting the overall metabolic status of an individual, offering a comprehensive view of obesity-related changes including the excretion of metabolites derived from various biological processes [30]. Unlike plasma and serum, urine is less affected by factors such as diet, medication, or circadian rhythms [31]. Metabolomics studies in adolescent populations are still limited. To gain a deeper understanding of obesity’s development, it is essential to validate previous research and examine metabolic changes in obese children who have not yet manifested disease symptoms.

Here, we examined the association of adolescent urinary metabolic signature with BMI z-scores to investigate the mechanisms of progression of childhood obesity at a metabolite level, which may aid in identifying adverse effects of obesity. This is the largest study of untargeted metabolomics in adolescents and is strengthened by longitudinal assessment. 

## 2. Materials and Methods

### 2.1. Study Recruitment and Design

**Figure 1:**
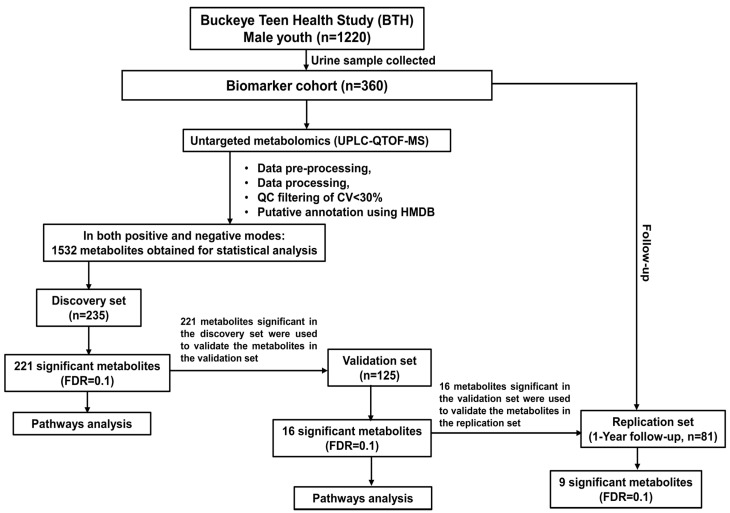
The flow diagram describes the discovery set, validation set, and replication set (1-year follow-up), data processing, statistical analysis, and a number of significant metabolites determined in each experiment that were used for pathway enrichment analysis.

A total of 1220 male youths were enrolled in the Buckeye Teen Health Study (BTH) from January 2015 to June 2016, a longitudinal cohort study focusing on lifestyle and tobacco use behaviors [32,33,34,35,36,37,38]. Out of this, a total of 360 subjects additionally provided urine samples as a “biomarker cohort” based on age, county, and date of sample collection (Figure 1). Eligible male youths were aged 11 to 16 years and lived in either urban Franklin County, Ohio, or 1 of 9 Appalachian Ohio counties. One year follow-up metabolomics study, referred to as a replication set, was conducted with eighty-one participants from the biomarker cohort (Figure 1). All participants in this study were informed in writing regarding collecting their samples for research aims and given the right to refuse such uses. Male youths provided consent, and their parents provided permission for them to enroll in the study. Exclusion criteria included any hearing or vision impairments or the inability to read and speak English. The baseline and one-year follow-up sessions were completed in person at participants’ homes or a mutually agreed-upon public location. The anthropometric measurements and data collection was conducted by a trained interviewer who resided in the same region as the participants. Data collected include demographic, family, and socioeconomic characteristics, as well as anthropometric measurements, such as height, body weight, and dietary records. BMI (kg/m^2^) or BMI percentile, and BMI z-score (https://zscore.research.chop.edu/calcbmi.php, accessed on 22 May 2022) were calculated based on the measured height and weight data. The questionnaire included both interviewer-administered and audio-administered items, depending on the level of sensitivity of the item to provide the boy’s privacy when their parents were around. Participants listened to these questions on a headset and responded on the computer without the assistance of the interviewer. The study protocol was approved by the Ohio State University Clinical Scientific Research Committee and Institutional Review Board (2014C0030).

### 2.2. Urine Sample Collection

Each study subject submitted a random single-void urine sample for analysis in a sterile container. Urine samples collected were shipped using an ice pack after freezing to OSU. Samples were stored at −80 °C for long-term storage until further analysis.

### 2.3. Reagents and Chemicals

All reagents and solvents were of HPLC grade. Formic acid, acetonitrile (ACN), 4-nitrobenzoic acid (4-NBA), and 13 C-labelled phenylalanine were purchased from Sigma-Aldrich (St. Louis, MO, USA) and water was purchased from Fisher Optima grade (Fisher Scientific, Waltham, MA, USA).

### 2.4. Urine Sample Preparation for Metabolomics

Urine was thawed at room temperature for 15 min and centrifuged for 10 min at 15,000 rpm at 4 °C. For positive mode sample analysis, the supernatant of the urine (20 µL) sample was diluted with water (180 µL) containing formic acid (0.1%) and 13 C-labelled phenylalanine (1.0 µM) as internal standard. For negative mode sample analysis, the supernatant of the urine (20 µL) sample was diluted with water (180 µL) containing formic acid (0.1%) and 4-NBA (2.0 µM) as internal standard. The final solution was vortexed for 30 s, transferred into HPLC vials, and placed into an autosampler tray for analysis.

### 2.5. UPLC–QTOF-MS Analysis

The metabolite separations of the urine sample were obtained on the Agilent 1290 Infinity Quaternary LC System (Agilent Technologies, Santa Clara, United States) using the ACQUITY UPLC HSS T3 column (2.1 × 100 mm, 1.8 μm). The injection volume was 1 μL, followed by a standard needle wash. The mobile phase (A) consisted of 100% water (H_2_O) with 0.1% formic acid and a mobile phase (B) consisted of 100% ACN with 0.1% formic acid with the following gradient elution. The flow rate was set as 0.5 mL/min and the gradient consisted of 100% A; 0–1.5 min, 0% B; 1.5–7.5 min, 50% B; 7.5–8.5 min, 95% B; 8.5–10 min, 95% B; 10–10.1 min, 0% B; and 10.10–12 min, 0% B. The column eluent (1 µL) was introduced directly into the mass spectrometer by electrospray. The autosampler tray temperature was set to 4 °C and the column temperature was 40 °C. The metabolic profiling analysis of the urine sample was conducted on an Agilent 6550 iFunnel Q-TOF LC/MS (Agilent Technologies, USA) with Dual Agilent Jet Stream Electrospray Ionization (Agilent Technologies), and its parameter was set as follows: for positive mode, dry gas temperature, 150 °C; dry gas flow, 18 L min^−1^; nebulizer pressure, 30 psig; sheath gas temperature, 300 °C; and sheath gas flow, 12 L min^−1^, and for negative mode, the instrument parameter settings are dry gas temperature, 200 °C; dry gas flow, 18 L min^−1^; nebulizer pressure, 35 psig; sheath gas temperature, 320 °C; and sheath gas flow, 12 L min^−1^. Mass spectrometry was performed in both positive-ion (ESI+) or negative-ion (ESI−) electrospray ionization mode with a capillary voltage of 4000 V and a sampling cone voltage of 65 V in both negative mode and positive mode. The scan range was adjusted to centroid mode using a scan rate of 3.00 spectra/second, and a mass range of 50–1700 *m*/*z*. 

A quality control (QC) sample was prepared by mixing an equal volume of a pooled urine sample of subjects containing 4-NBA (2.0 µM) and 13 C-labelled phenylalanine (1.0 µM) as internal standard, which was then aliquoted into small vials. The pooled QC samples were used to condition the analytical platform at the beginning of the run, placed as every tenth sample, and analyzed periodically after 10 sample runs to check the performance of the analytical system in terms of retention times, accurate mass measurements, and signal intensities.

### 2.6. Data Analysis

After completing the metabolomics run on the instrument, we processed the raw data from both the discovery and validation set together. The raw data (.d file) from the ultra-performance liquid chromatography-quadrupole-time-of-flight mass spectrometry (UPLC-QTOF-MS) instrument were converted to mzML files using the MS convert program from ProteoWizard (https://proteowizard.sourceforge.io/, accessed on 22 May 2022). In the resulting mzML files, intensity and *m*/*z* values were stored as 32-bit floating points with zlib compression, and the “vendor peak picking” option was selected to convert data to centroid mode. The mzML files were imported into the R statistical computing environment using the R package XCMS [39]. XCMS was used for data processing including retention time alignment, peak detection, peak grouping, and peak filling to obtain a sample-feature matrix. Retention time alignment was performed using the ObiWarp algorithm. Each sample was aligned against the pooled QC sample in both EIC+ and EIC− modes. Peak detection was performed using the CentWave algorithm (parameters: peak width = 2–30 s, signal-to-noise ratio = 3, mzdiff = −0.005, integration method = 1, prefilter = 3 peaks, 10 intensity). Peak grouping was performed using the peak density method (parameters: minFraction = 0, minSamples = 2, bw = 5, binSize = 0.015). Integration of signal from peaks missing after grouping peaks across samples was performed with the fillChromPeaks function and default parameters (expandMz = 0, expandRt = 0, ppm = 0, fixedMz = 0, fixedRt = 0). The resulting data contained a set of features corresponding to peaks which were found in multiple samples each with an approximate *m*/*z* and retention time. The matrix of intensity values by feature and sample was used for further analysis. During the profiling, the analytical robustness of UPLC-QTOF-MS QC samples was repeatedly analyzed. Metabolite features that were missing in more than one pooled QC sample or showing a large coefficient of variation (CV) in the pooled QC samples greater than 30% were discarded as unreliable before conducting the statistical analysis. Intensity values were normalized by the mass spectrometry total usable signal (MSTUS). Briefly, an MSTUS value was calculated by summing the intensity of all features, excluding those features which were either found in less than half of the samples, in the bottom 20th percentile of peak intensities, in more than 80% of samples, or were in the 90th percentile in at least one but less than 10% of samples. Feature intensities for each sample were then multiplied by a scaling factor calculated as the median MSTUS value over the sample MSTUS value. Missing feature intensities after peak filling were then imputed as half of the minimum value by feature [40]. 

### 2.7. Statistical Analysis

Before metabolomics analyses, patients were randomized into a modeling set for the discovery (n = 235), validation (n = 125), and one-year follow-up as replication sets (n = 81) (Figure 1). 

JMP Pro 15 (100 SAS Campus Drive Cary, NC, USA) was used for statistical analysis. Data of normal distribution are expressed as mean ± SD. Measurement data of non-normal distribution are expressed as median (interquartile range). Before statistical analyses, urinary metabolomics peak intensity data were log10-transformed. Preliminarily annotated metabolites present in both cohorts were analyzed. The Partek Genomics suite was used for the principal component analysis [41]. Linear regression analysis models were used to estimate the associations of metabolic features with a BMI z-score in the discovery and validation set, after adjusting for age, race, and total energy intake (kcal). In the present study, smoking status was less than 4.2%, and smoking status and socioeconomic status were initially included in the models but were removed due to the lack of significance of the effect (Supplement Tables S4 and S5).

After pooled QC filtering of CV < 30% and performing putative annotation, a total of 1532 metabolite features were obtained in both positive and negative modes. These features were then subjected to linear regression analysis in the discovery set. Subsequently, 221 features that exhibited significant association with BMI Z-score in the discovery set were selected for further investigation of their association with BMI Z-score in the validation set.

Correction for multiple comparisons was performed using a false discovery rate (FDR) [42] corrected alpha of ≤0.1. Graph Pad Prism version 8.4 was used to produce the volcano plots. To identify pathways that may have been perturbated in adolescent children, metabolic pathway analysis was performed using Metaboanalyst 5.0 [43,44] (http://www.metaboanalyst.ca/). Metabolites that showed an association with BMI Z-score in the discovery set and validation set were queried against the human metabolome database (HMDB, www.hmdb.ca, accessed on 22 May 2022). The list of putative metabolites generated by HMDB was imported into Metaboanalyst and mapped based on the Kyoto Encyclopedia of Genes and Genomes (KEGG). Pathway enrichment ratio values > 1.0 and a *p*-value < 0.05 was considered to define a perturbed pathway. The top 25 pathways were reported as the most perturbated pathways.

## 3. Results

### 3.1. Characteristics of Study Subjects

The baseline characteristics of the discovery, validation set, and replication set study participants are shown in Table 1. In total, 360 study participants were recruited into the BTH study for urine metabolomics, including 235 in the discovery set and 125 in the validation set. The average age (mean ± SD) of the study participant was 14.80 ± 1.42 years, 14.84 ± 1.31 years, and 16.08 ± 1.20 years in the discovery set, validation set, and replication set study participants, respectively. The age and height of the study participants in the discovery and validation sets were similar, but the weight of the study participants in the discovery (158.50 lb.) and validation set (149.85 lb.) slightly differed. As expected, we observed an increase in weight (170.64 lb.) in the replication set study participants. The average BMI (Kg/m^2^) of the study participants in the discovery set, validation set, and replication set study were 24.64 ± 7.13, 23.28 ± 6.01, and 25.16 ± 6.91, respectively. At baseline, 31.49 and 22.40% of the study participants were obese (Table 1). 

### 3.2. Untargeted Metabolic Profiling of Urine by UPLC-QTOF-MS

In the UPLC-QTOF-MS dataset, a total of 4596 features were detected in the positive mode (2537) and negative mode (2059) after pooled QC filtering of CV < 30%. Further, after annotation using the Human Metabolome Database (HMDB) (https://hmdb.ca/spectra/ms/search), we obtained that 1532 metabolite features in both positive mode and negative mode were used for statistical analysis. The overall composition of the distributions of these metabolites via principal component analysis found complex underlying correlations; in particular, the first principal component explained 17.7% of the total variation in the levels of these 1532 metabolites (Supplementary Figures S1 and S2A,B).

### 3.3. Association of Metabolites with BMI Z-Score in Discovery and Validation Set, and Changes in BMI at the 1-Year Follow-Up

**Figure 2:**
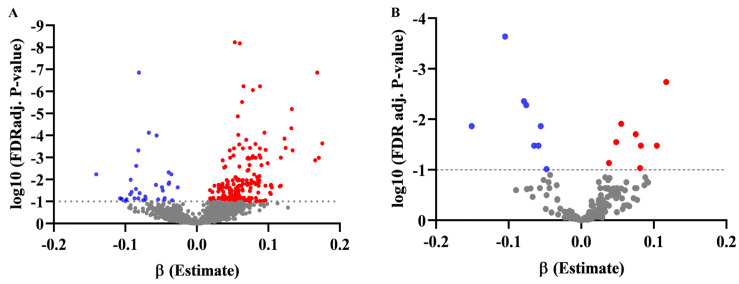
The association between metabolites and BMI Z−score. Volcano plot: each point on the volcano plot was based on estimates of beta (β) from linear regression analysis on the x−axis versus the log10 FDR adj. *p*−value on the y−axis of the putatively identified metabolites. (**A**) Volcano plot of association of metabolites with BMI z−score in the discovery set; (**B**) volcano plot of association of metabolites with BMI z−score in the validation set (red dot for a positive association and blue dot for a negative association, horizontal dotted lines set false discovery rate 10% corrected *p*−value).

In the discovery set, a total of 221 features were significantly associated with BMI z-score after adjustment for covariables (age, race, and total energy intake) and multiple comparisons (fdr ≤ 0.1). The estimates of the association of metabolites with BMI z-score and their corresponding *p*-value after multiple test corrections are displayed in Figure 2A, Supplement Table S1, and the top 30 are displayed in Table 2. Out of 221 significant features associated with BMI z-score, 32 metabolites are negatively associated with BMI z-score, and 189 metabolites were positively associated with BMI z-score (Supplement Table S1)**.**

**Figure 3:**
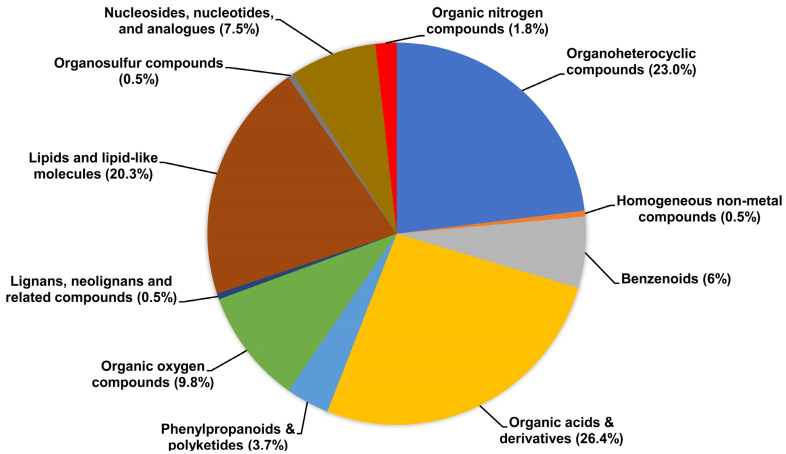
A pie chart summarizing the range and classes of compounds (super-class annotated by HMDB) demonstrates a significant association of metabolites with BMI z-score in the discovery set of urine samples from adolescents.

These 221 putatively identified metabolites in discovery sets refer to 11 chemical taxonomy super-class and 28 sub-class families (Supplemental Table S1)**.** Out of these 11 super-class taxonomies, the most perturbated super-class of compounds belonged to organic acids and their derivatives (amino acids, peptides, and analogues), lipids and lipid-like molecules, organic oxygen compounds carbohydrates, and carbohydrate conjugates), organoheterocyclic compounds (pyridines, indol, imidazoles, indolyl carboxylic acids, and derivatives), nucleosides, nucleotides, and analogues (Figure 3, Supplement Table S1).

In the validation set analysis, we assessed 221 significant metabolites from the discovery set, and after controlling for covariates and multiple comparisons, we found that 16 of the 221 metabolites remained significantly associated with BMI z-score in the validation set (FDR = 0.1) (Figure 2B, Table 3, Supplement Table S1). Among 16 significant metabolites associated with a BMI Z-score in the validation set, 8 metabolites were negatively associated and 8 were positively associated (Figure 2B, Table 3). The positive change in BMI in the replication set (1 year of follow-up) indicated that among the 16 replicated in the validation set, 9 were also found to be correlated with the positive change in BMI (Table 4). 

### 3.4. Differential Mapping of Metabolites in Pathway Analysis

**Figure 4:**
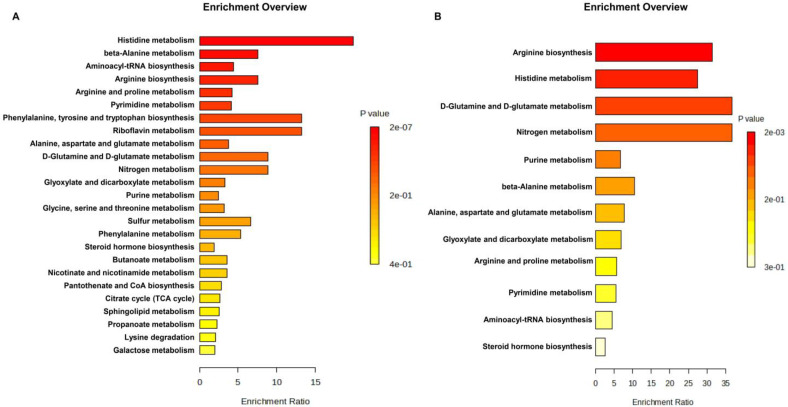
Pathway analysis as generated by MetaboAnalyst 5.0 software package for metabolites significantly expressed in adolescent children. The enrichment ratio is calculated as the number of hits within a particular metabolic pathway divided by the expected number of hits. Metabolite set enrichment analysis (MSEA). Top perturbed pathways are shown. (**A**) Metabolic pathway analysis using the list of 221 significant features in the discovery set, and (**B**) a list of 16 significant features in the validation set. The color depth and column length indicate the disturbance degree of the pathway.

To explore the metabolic pathways that potentially contribute to obesity progression in adolescent children, we carried out a metabolomic pathway enrichment analysis using MetPA (www.metaboanalyst.ca) with 221 significant features from the discovery set and 16 significant features from the validation set. *p*-values < 0.05 and enrichment ratios > 0.1 were considered to indicate highly influential pathways. For the discovery set, pathway enrichment results showed that 25 metabolic pathways were identified in KEGG. The highest enrichment ratios were obtained for the histidine metabolism pathways, beta-Alanine metabolism, aminoacyl-tRNA biosynthesis, arginine biosynthesis, arginine and proline metabolism, and pyrimidine metabolism are the most enriched metabolic pathways, as shown in Figure 4A, and detailed pathway enrichment results are displayed in Supplement Table S2. Similarly, in the validation set, arginine biosynthesis, histidine metabolism, D-glutamine and D-glutamate metabolism, and nitrogen metabolism were the most significantly enriched metabolic pathways (Figure 4B and Supplement Table S3). In both instances of pathway enrichment analysis, arginine biosynthesis and histidine metabolism were the most enriched metabolic pathways. 

## 4. Discussion

The purpose of the study was to identify the metabolomic signature of obesity in adolescents. Prior studies in the field of metabolomics have predominantly focused on characterizing metabolites linked to childhood obesity or BMI in contrast to non-obese subjects [27,45,46,47,48]. Obesity mainly classifies adolescents as normal, overweight, or obese based on their BMI percentile [15,16]. As children grow up, the measurement of body mass index (BMI) requires adjustment for age and gender [14]. However, the BMI z-score as a continuous variable, which is age and gender-specific in children, is considered a more suitable metric for determining body mass index (BMI) than the conventional BMI measurement and provides a better estimation of an individual’s adiposity status [17]. 

The present cross-sectional study demonstrated the perturbation of the urinary metabolites associated with BMI z-score in male adolescents and replicated results in the longitudinal analysis for changes in BMI. Additionally, metabolomic signatures were identified with the potential to examine metabolic health in adolescents. To our knowledge, this is the first longitudinal study to relate positive change in BMI to the urine metabolome in adolescents. In this study, 1532 putatively annotated features were used to study the association analysis with BMI Z-score in adolescents. Following the adjustment of age, race, and total energy intake, 221 metabolites were identified as exhibiting a robust association with BMI z-score within the discovery set. Out of 221 lists of significant features from the discovery set, 16 metabolites were found to be significantly associated with the BMI z-score in the validation dataset in adolescents. Obesity is a serious and growing health problem that affects people of all ages all over the world, including children. In the past, research on metabolomic biomarkers in obesity has largely focused on targeted metabolomics and a specific group of compounds rather than evaluating a large number of metabolites at the same time [49,50,51,52]. However, developing evidence indicates that not only absolute metabolite levels of specific compounds are important, but their relationship with other metabolites (profiles) and pathways play an important role in the biology of metabolism [53]. Therefore, it is crucial to simultaneously investigate a larger number of metabolites to arrive at a more accurate etiologic picture. Our findings highlight the presence of changes in the urine metabolome associated with positive changes in BMI in adolescence. We observed that the six urinary metabolites (3′-sialyllactose, formiminoglutamic acid, 4-hydroxyproline, citrulline, inosine) showed a positive association with positive change in BMI and three metabolites (glycylproline, 4-vinylsyringol) showed a negative association with positive change in BMI. Accumulated evidence showed that the perturbated metabolites are amino acids and carbohydrates. 

In several adolescent studies, the relationship between the metabolome and obesity has been thoroughly described [1,17,21,27]. The present study's finding is consistent with previous findings and most of the metabolites strongly influencing metabolic BMI are documented in the present study. In the study by Cirulli et al. (2019), which included 1969 individuals from the TwinsUK cohort, 49 metabolites showed the strongest associations with BMI out of a total of 650 metabolites, and a majority of the 49 metabolites were identified as significant predictors of BMI. These included glutamate, asparagine, leucine, N2,N2-dimethylguanosine, and kynurenate, among others [17]. Thus, only 7.54% of metabolites were replicated across multiple studies [17], a finding similar to that of the present study, where 7.27% of metabolites were replicated in validation sets. Similarly, Sohn et al. (2022) investigated metabolomic signatures associated with weight control interventions in children with obesity using untargeted metabolomics in plasma samples and observed 12 metabolites were significant at both time points including asparagine, glutamine, O-acetylcarnitine, and most perturbated metabolic pathways was D-glutamine and glutamate metabolism and arginine biosynthesis [1]. The present study results also find D-glutamine and glutamate metabolism (L-Glutamine) and arginine biosynthesis (L-Glutamine) were the most perturbated metabolic pathways. Another study by Cho et al. (2017) included non-obese (n = 91) and obese (n = 93) adolescents from both sexes and also conducted untargeted and targeted urinary metabolomics. Inflammation-related metabolites were identified with strong predictive power to distinguish obese and non-obese groups, and acylcarnitines (hexanoylcarnitine), amino acid (glutamine, asparagine), amines (carnosine), glycerophospholipids, and sphingolipids were significantly high in obese adolescents [27]. In our study, we found 2-hexanoylcarnitine, L-glutamine, carnosine, and hydroxyprolyl-asparagine are associated with BMI Z-score. Obesity is associated with increased acylcarnitine levels in blood plasma sample of Hispanic children, e.g., propionyl-, butyryl-, hexanoyl-, stearoyl-, and oleoylcarnitine [46], and in adolescence, e.g., propionyl-, 2-methylbutyryl-, isovaleryl-, and isobutyrylcarnitine [54]. In this study, 2-hexenoylcarnitine was associated with the BMI z-score. Acylcarnitines play an important role in many cellular energy metabolism pathways, e.g., transporting acyl groups (organic acids and fatty acids) from the cytoplasm to the mitochondria for the production of energy [55,56]. Papandreou et al. (2021) reported acylcarnitine metabolites (hexanoylcarnitine, hexadecenoylcarnitine) were associated with body fat% [57], indicating that higher body fat correlates with upregulated beta-oxidation of fatty acids. Acylcarnitines are important biomarkers in metabolic studies such as metabolic disorders, cardiovascular diseases, diabetes [56], kidney cancer [58], and hepatocellular carcinoma [59]. Observed elevated levels of carnitine metabolites in adolescents require particular attention and may be a target for obesity management to prevent the development of disease complications.

As previously reported by Brachem et al. (2020), the urinary level of glucuronide of C10H18O2 (12) was positively associated with BMI and body fat in adolescents [23]. In addition, in another study, Tchernof et al. (1997) reported the plasma level of androstane-3α,17β-Diol glucuronide concentration was significantly higher in overweight men [60]. The liver is the primary site of altered glucuronidation, with the help of the UDP-glucuronyl transferase enzyme in conjugation with glucuronate, resulting in the removal of toxic substances, drugs, or other xenobiotics [61,62]. Glucuronides in adipose tissue have been shown to demonstrate higher activity in obese individuals [60,63]. In the current study, N-(2-hydroxyphenyl) acetamide glucuronide was found to be positively associated with BMI Z-score and positive change in BMI in adolescents. The current study observation is consistent with previous studies in that the glucouronide product is associated with obesity [64]. The mechanism of glucouronide products and their relationship with obesity is currently unknown.

Increased adiposity is also associated with changes in amino acid metabolism [47]. In the etiology of obesity and diabetes mellitus, amino acids (AAs) are emerging as a new class of potent molecules. Amino acids (AA) are the building blocks of proteins and play essential roles in gene expression, cell signaling, inflammatory responses, metabolism, oxidative stress, and detoxification [65]. Yamakado et al. reported that the change in the amino acid profile was closely related to the development of metabolic complications such as insulin resistance, diabetes, and visceral fat accumulation [66]. We observed a negative association of BMI z-score with amino acids and their derivatives such as glycylproline, L-glutamine, hydroxyprolyl-asparagine, and galactosylhydroxylysine. In contrast, formiminoglutamic acid, 4-hydroxyproline, and citrulline were positively associated with BMI Z-score. These findings are consistent with those of previous research, where obesity was associated with higher levels of amino acids such as lysine, tryptophan, cystine, and glutamate, but lower levels of asparagine, glutamine, glycine, and serine in Japanese adults [49]. In comparison to children of normal weight, children with obesity had reported higher serum levels of phenylalanine, proline, histidine, and lysine and lower serum levels of glutamine [67]. Citrulline is associated with the urea cycle. A previous study reported a decrease in citrulline in obese adolescents [27,46,67]. While the present study results are inconsistent, we found citrulline is positively associated with BMI z-score and a positive change in BMI in adolescents. Moreover, branched-chain amino acids (BCAAs) and aromatic amino acids have previously been proposed as biomarkers of metabolic syndrome [50]. BCAAs promote protein synthesis and turnover, signaling pathways, and metabolism of glucose. Oxidation of BCAAs may increase fatty acid oxidation and play a role in obesity. In the present study, we also observed a significant positive association of 4-hydroxyproline with BMI z-score and a positive change in BMI in a follow-up study in adolescents. Increasing evidence has been reported that 4-hydroxyproline may play a significant role in protecting mammalian cells from oxidative stress and injury [68]. Additionally, 4-hydroxyproline enhances human nutrition and health, including metabolic, immune, and cardiovascular health [69]. 4-hydroxyproline and carnosine from dietary sources are beneficial for preventing and treating obesity, cardiovascular dysfunction, and aging-related disorders, as well as inhibiting tumorigenesis in children and adults [69]. Our results are consistent with Cho et al. (2017), where carnosine a dipeptide synthesized in the body from β-alanine and L-histidine was found to decrease in obese adolescents [27]. In the current study, we observed galactosylhydroxylysine was negatively associated with BMI Z-score. A study conducted urine metabolic profiling in normal-weight young men and obese men with hyperlipidemia. It was observed that glucosylgalactosyl hydroxylysine along with eight other metabolites has a significant impact on the development and manifestation of obesity-related disorders [70]. 

Glycylproline, a dipeptide composed of glycine and proline, is considered a building block for proteins. Glycine is also required for multiple metabolic pathways. Several studies reported the level of glycine was found to decrease in children with obesity in plasma samples [46,52]. Similarly, Wahl et al. (2012) reported the level of proline decreased in children with obesity [47]. However, Short et al. investigated plasma profiles of amino acid-related metabolites among Indian American adolescents and observed higher levels of proline in obese children as compared to normal-weight children [67]. In the present study, glycine-proline is negatively associated with BMI z-score and a positive change in BMI after a 1-year follow-up. In vitro and in vivo studies reported cyclic glycine-proline (cGP) mediates the homeostasis of insulin-like growth factor (IGF)-1 function and the cGP/IGF-1 ratio, which determines IGF-1 bioactivity. Plasma IGF-1 is predominantly inactive and slightly related to obesity and hypertension in humans [71]. Glycine and proline are non-essential amino acids (NEAAs) that play a crucial role in nutrition serving and are closely related to the development of tumors. Glycine deficiency reduces the synthesis of glutathione (GSH) and enhances reactive oxygen species (ROS) production. A decline in GSH levels and accumulation of ROS promote lipid peroxidation ultimately leads to tumor suppression through ferroptosis-mediated mechanisms [72]. Formiminoglutamic acid is an intermediate metabolite in the degradative conversion of histidine to glutamic acid [73]. Formiminoglutamic acid is not directly related to obesity, but increased levels of urinary formiminoglutamic acid are associated with a deficiency of folic acid and vitamin B12, which may lead to liver disease [74], CVD [75], and heart disease [76]. In this study, formiminoglutamic acid is positively associated with BMI z-score and a positive change in BMI. The pathway analysis report suggests which metabolic pathways might be altered in individuals with different BMI z-scores and positive changes in BMI. The most perturbated metabolic pathway in the current study is arginine biosynthesis. Arginine is synthesized from citrulline and L-glutamine. Recent findings from human and animal-based research suggested that arginine plays a crucial role in modulating the metabolism of energy substrates [77]. Arginine metabolism stimulates the expression of specific genes in adipose tissue, potentially increasing fatty acid and glucose oxidation [78]. Previous investigations have exhibited that the intake of L-arginine can improve endothelial function, insulin secretion and sensitivity, and inflammation, all of which are interconnected with obesity-induced ailments such as type 2 diabetes mellitus and cardiovascular diseases [79,80,81].

Our study has some limitations. We evaluated a sample of individuals mainly consisting of only male adolescents with normal/overweight/obesity, which could limit the generalizability of our results to other populations and girls. Additionally, we provide no information about the association of obesity and metabolomic features in adults, when BMI is generally more stable and not impacted by changes in hormones (e.g., growth hormones). Second, in our study, we used BMI z-score as a criterion for obesity, rather than body-fat and waist circumference, which can be a more accurate parameter of adiposity. Third, since metabolites were only measured at a one-time point (baseline) and one-year follow-up with a limited sample size (n = 81), we could not provide any data regarding the stability of the metabolome associated with BMI z-score and positive change in BMI over longer times. Fourth, this study did not positively identify the metabolites and apply a targeted approach so that the putative identifications may not be correct. Fifth, we used random urine samples for metabolomics analysis and variability in the metabolite content may be a limitation.

The present study has notable strengths. This current study focuses on adolescent males, capturing what features remain associated with obesity over time. It incorporates both cross-sectional and longitudinal analysis. Replication was enhanced by using a discovery and validation set, which was then applied to longitudinal analysis. This increases the confidence of true findings. Reasons why the sampling at two time points is not concordant could be due to differences in lifestyle at the different time points, changing metabolomic profiles as the participants age, and/or false negatives in spite of multiple comparison corrections. Another strength is the use of the z-score, which is better reflective of future health status as it accounts for age and gender. Additionally, comprehensive untargeted metabolome profiling was performed on UPLC-Q-TOF-MS metabolomic platforms, which provides maximum coverage as compared to targeted metabolomics and another approach to qualitatively analyze a wide range of metabolites. It also helps in the identification of new metabolites associated with obesity and obesity-related complications. 

## 5. Conclusions

The present study assessed the metabolomic profile using an untargeted metabolomics approach and most of the significant metabolic features observed in the current study are consistent with the adolescent study. The current study indicates histidine metabolism as a key mechanism related to obesity. The novel whole-metabolome approach evidenced several biomarkers related to obesity, most of which are carboxylic acid derivatives (glycylproline, 4-hydroxyproline L-glutamine, citrulline, galactosylhydroxylysine), carnitines (2-hexenoylcarnitine), lipids (N-(2-hydroxyphenyl) acetamide glucuronide), carbohydrates (3′-sialyllactose), while several others have been evidenced in the discovery set. These metabolites may directly or indirectly contribute to the development of obesity, particularly its effects, which can be studied in more detail in mechanistic studies. Obesity is a multifaceted and complex health concern that arises from a combination of genetic, behavioral, social, and environmental factors. While biological factors certainly play a role in an individual’s susceptibility to obesity, the environment in which we live greatly influences our food choices, physical activity levels, and overall lifestyle. Even though the use of metabolomics in childhood obesity research is still in its early stages, the identified metabolites have provided additional insight into the pathogenesis of some obesity-related diseases. Furthermore, future research should attempt to replicate our findings in a different adolescent population and extend the analysis to a longitudinal design to better understand the possible correlation of body composition with urine metabolome.

## Figures and Tables

**Table 1:** Characteristics of the study participants.

Parameter	Discovery Set (N = 235)	Validation Set (N = 125)	Replication Set (N = 81)
Age (Year) (Mean ± SD) Range	14.80 ± 1.42 (11.14–16.99)	14.84 ± 1.31 (11.06–16.99)	16.08 ± 1.20 (12.9–18)
Height (Inch) (Mean ± SD) Range	66.82 ± 4.10 (56.00–75.67)	66.97 ± 3.87 (57.25–75.25)	68.88 ± 3.18 (60–75)
Weight (lb) (Mean ± SD) Range	158.50 ± 54.36 (72.93–334.13)	149.85 ± 45.14 (78.2–304.6)	170.64 ± 50.59 (79–320)
BMI (Kg/m^2^) (Mean ± SD) Range	24.64 ± 7.13 (15.3–49)	23.28 ± 6.01 (15.5–44.10)	25.16 ± 6.91 (14.4–45.9)
BMI percentile (Mean ± SD) Range	68.2 ± 30.43 (2–99)	66.54 ± 30.39 (2–99)	68.82 ± 29.11 (1–99)
BMI z-score (Mean ± SD) Range	0.82 ± 1.23 (−2.07–3.01)	0.60 ± 1.18 (−2.04–2.84)	0.78 ± 1.23 (−3.06–2.96)
Positive change in BMI (Kg/m^2^) Mean/Median (Range)	---	---	0.54/0.6 (−11.6–5.8)
Obesity (n (%)) Underweight (<5 percentile) Healthy weight (5 to <85 percentile) Overweight (>85 to <95 percentile) Obese (>95 percentile)	3 (1.28%) 129 (54.89%) 29 (12.34%) 74 (31.49%)	4 (3.20%) 70 (56.00%) 23 (18.40%) 28 (22.40%)	3 (3.70%) 47 (58.02%) 12 (14.81%) 19 (23.46%)
Race (n (%)) White Black Hispanic Multiracial Others	176 (74.47%) 36 (15.35%) 8 (3.39%) 12 (5.08%) 4 (1.69%)	99 (79.20%) 13 (10.40%) 4 (3.20%) 7 (5.60%) 2 (1.60%)	58 (71.60%) 13 (16.05%) 4 (4.94%) 5 (6.17%) 1 (1.23%)
County (n (%)) Franklin Non-Franklin	121 (51.49%) 114 (48.51%)	64 (51.20%) 61 (48.80%)	44(54.32%) 37(45.68%)
Total energy intake (kcals) (Mean/Median) Range	1883.84/1745.54 (204.05–5209.14)	1937.77/1873.41 (462.5–5549.48)	1778.03/1507.12 (236.91–4589.2)

Abbreviations: BMI, body mass index; SD, standard deviation.

**Table 2:** Top 30 metabolic features associated with BMI z-score in the discovery set of samples.

ID	Metabolite	Mode	MZ	RT	Adduct	HMDB ID	Super-Class	Class	Sub-Class	Discovery Set		
										Estimate (95%CI)	*p*-Value	FDR adj
neg_FT17207	3′-Sialyllactose	Neg	632.2	0.67	M − H	HMDB0000825	Organic oxygen compounds	Organooxygen compounds	Carbohydrates and carbohydrate conjugates	0.05 (0.03–0.06)	<0.0001	5.82 × 10^−9^
pos_FT29354	3′-Sialyllactose	Pos	656.2	0.67	M + Na	HMDB0000825	Organic oxygen compounds	Organooxygen compounds	Carbohydrates and carbohydrate conjugates	0.06 (0.04–0.07)	<0.0001	6.56 × 10^−9^
pos_FT04847	Estrone sulfate	Pos	176.06	0.58	M + 2H	HMDB0001425	Lipids and lipid-like molecules	Steroids and steroid derivatives	Sulfated steroids	−0.08 (−0.1–−0.05)	<0.0001	1.38 × 10^−7^
pos_FT13024	N-Ribosylhistidine	Pos	288.11	0.53	M + H	HMDB0002089	Organic acids and derivatives	Carboxylic acids and derivatives	Amino acids, peptides, and analogues	0.16 (0.11–0.21)	<0.0001	1.38 × 10^−7^
pos_FT04858	Citrulline	Pos	176.1	0.78	M + H	HMDB0000904	Organic acids and derivatives	Carboxylic acids and derivatives	Amino acids, peptides, and analogues	0.06 (0.04–0.08)	<0.0001	5.77 × 10^−7^
neg_FT15762	PA(22:5(4Z,7Z,10Z,13Z,19Z)-O(16,17)/2:0)	Neg	539.24	6.53	M − H	HMDB0266570				0.08 (0.06–0.11)	<0.0001	5.77 × 10^−7^
pos_FT27056	Tetrahydroaldosterone-3-glucuronide	Pos	563.24	6.57	M + Na	HMDB0010357	Lipids and lipid-like molecules	Steroids and steroid derivatives	Steroidal glycosides	0.07 (0.05–0.1)	<0.0001	8.64 × 10^−7^
neg_FT01967	Citrulline	Neg	174.08	0.81	M − H	HMDB0000904	Organic acids and derivatives	Carboxylic acids and derivatives	Amino acids, peptides, and analogues	0.13 (0.08–0.17)	<0.0001	6.28 × 10^−6^
neg_FT15806	Cortolone-3-glucuronide	Neg	541.26	6.18	M − H	HMDB0010320	Lipids and lipid-like molecules	Steroids and steroid derivatives	Steroidal glycosides	0.05 (0.03–0.07)	<0.0001	1.34 × 10^−5^
neg_FT01921	Formiminoglutamic acid	Neg	173.05	1.31	M − H	HMDB0000854	Organic acids and derivatives	Carboxylic acids and derivatives	Amino acids, peptides, and analogues	0.13 (0.08–0.18)	<0.0001	4.66 × 10^−5^
pos_FT04673	Glycylproline	Pos	173.09	0.75	M + H	HMDB0000721	Organic acids and derivatives	Carboxylic acids and derivatives	Amino acids, peptides, and analogues	−0.06 (−0.09–−0.04)	<0.0001	7.46 × 10^−5^
neg_FT02197	Galactitol	Neg	181.07	1.81	M − H	HMDB0000107	Organic oxygen compounds	Organooxygen compounds	Carbohydrates and carbohydrate conjugates	0.09 (0.05–0.13)	<0.0001	7.46 × 10^−5^
pos_FT27104	Cortolone-3-glucuronide	Pos	565.26	6.1	M + Na	HMDB0010320	Lipids and lipid-like molecules	Steroids and steroid derivatives	Steroidal glycosides	0.05 (0.03–0.08)	<0.0001	9.30 × 10^−5^
pos_FT09630	4-Vinylsyringol	Pos	243.09	0.73	M + H	HMDB0301746	Phenylpropanoids and polyketides	Stilbenes		−0.05 (−0.07–−0.03)	<0.0001	9.96 × 10^−5^
neg_FT07183	Fludiazepam	Neg	301.05	2.36	M − H	HMDB0015513	Organoheterocyclic compounds	Benzodiazepines	1,4-benzodiazepines	0.12 (0.07–0.17)	<0.0001	1.38 × 10^−4^
neg_FT02892	Adipoylglycine	Neg	202.07	3.23	M − H	HMDB0240731	Organic acids and derivatives	Carboxylic acids and derivatives	Amino acids, peptides, and analogues	0.06 (0.04–0.09)	<0.0001	1.57 × 10^−4^
neg_FT11170	6-Hydroxymelatonin glucuronide	Neg	389.18	6.34	M + Cl	HMDB0060786	Organic oxygen compounds	Organooxygen compounds	Carbohydrates and carbohydrate conjugates	0.17 (0.1–0.24)	<0.0001	2.29 × 10^−4^
neg_FT15862	N-Acetylgalactosaminyl lactose	Neg	544.18	0.71	M − H	HMDB0041622	Organic oxygen compounds	Organooxygen compounds	Carbohydrates and carbohydrate conjugates	0.08 (0.04–0.11)	<0.0001	2.39 × 10^−4^
neg_FT02148	3-Chlorotyrosine	Neg	180.06	1.81	M + Cl	HMDB0001885	Organic acids and derivatives	Carboxylic acids and derivatives	Amino acids, peptides, and analogues	0.05 (0.03–0.08)	<0.0001	2.55 × 10^−4^
pos_FT18573	Cephalexin	Pos	370.08	5.82	M + Na	HMDB0014707	Organoheterocyclic compounds	Lactams	Beta lactams	0.12 (0.07–0.17)	<0.0001	3.66 × 10^−4^
neg_FT08222	Dihyroxy-1H-indole glucuronide I	Neg	324.07	3.91	M − H	HMDB0059997	Organic oxygen compounds	Organooxygen compounds	Carbohydrates and carbohydrate conjugates	0.07 (0.04–0.1)	<0.0001	3.66 × 10^−4^
neg_FT14008	3-alpha-hydroxy-5-alpha-androstane-17-one 3-D-glucuronide	Neg	465.24	7.84	M − H	HMDB0010365	Lipids and lipid-like molecules	Steroids and steroid derivatives	Steroidal glycosides	0.06 (0.03–0.09)	<0.0001	3.84 × 10^−4^
neg_FT14066	Clozapine glucuronide	Neg	467.19	6.92	M + Cl	HMDB0060901	Organic oxygen compounds	Organooxygen compounds	Carbonyl compounds	0.08 (0.05–0.12)	<0.0001	3.84 × 10^−4^
neg_FT01695	Quinolinic acid	Neg	166.01	1.17	M − H	HMDB0000232	Organoheterocyclic compounds	Pyridines and derivatives	Pyridinecarboxylic acids and derivatives	0.05 (0.02–0.07)	<0.0001	3.84 × 10^−4^
neg_FT08456	Hydroxytyrosol 3′-glucuronide	Neg	329.08	3.78	M − H	HMDB0240531	Organic oxygen compounds	Organooxygen compounds	Carbohydrates and carbohydrate conjugates	0.09 (0.05–0.13)	<0.0001	4.50 × 10^−4^
neg_FT01863	Glycylproline	Neg	171.07	0.91	M − H	HMDB0000721	Organic acids and derivatives	Carboxylic acids and derivatives	Amino acids, peptides, and analogues	−0.08 (−0.11–−0.04)	<0.0001	4.68 × 10^−4^
neg_FT09551	5-Caffeoylquinic acid	Neg	353.08	6.84	M − H	HMDB0240477	Organic oxygen compounds	Organooxygen compounds	Alcohols and polyols	0.13 (0.07–0.19)	<0.0001	4.68 × 10^−4^
neg_FT00341	(R)-3-Hydroxyisobutyric acid	Neg	103.04	2.02	M − H	HMDB0000336	Organic acids and derivatives	Hydroxy acids and derivatives	Beta hydroxy acids and derivatives	0.04 (0.02–0.06)	<0.0001	4.75 × 10^−4^
neg_FT02346	1-(Malonylamino)cyclopropanecarboxylic acid	Neg	186.04	3.48	M − H	HMDB0031700	Organic acids and derivatives	Carboxylic acids and derivatives	Amino acids, peptides, and analogues	0.08 (0.04–0.12)	<0.0001	7.96 × 10^−4^
neg_FT14465	11-beta-Hydroxyandrosterone-3-glucuronide	Neg	481.24	6.63	M − H	HMDB0010351	Organoheterocyclic compounds	Indoles and derivatives	Hydroxyindoles	0.05 (0.02–0.07)	<0.0001	8.07 × 10^−4^

Model: metabolites = age + race + BMI z-score + total energy intake. Abbreviations: ID, metabolic feature identification; MZ, mass-charge; RT, retention time; HMDB, human metabolome database.

**Table 3:** List of significant metabolic features associated with BMI z-score in the validation set of samples.

ID	Metabolite	Mode	MZ	RT	Adduct	HMDB ID	Super-Class	Class	Sub-Class	Validation Set		
										Estimate (95%CI)	*p*-Value	FDR adj
pos_FT04673	Glycylproline	Pos	173.09	0.75	M + H	HMDB0000721	Organic acids and derivatives	Carboxylic acids and derivatives	Amino acids, peptides, and analogues	−0.105 (−0.145–0.064)	<0.0001	2.29 × 10^−4^
neg_FT01967	Citrulline	Neg	174.08	0.81	M − H	HMDB0000904	Organic acids and derivatives	Carboxylic acids and derivatives	Amino acids, peptides, and analogues	0.117 (0.065–0.168)	<0.0001	0.002
pos_FT09630	4-Vinylsyringol	Pos	243.09	0.73	M + H	HMDB0301746	Phenylpropanoids and polyketides	Stilbenes		−0.079 (−0.117–0.041)	<0.0001	0.004
neg_FT01863	Glycylproline	Neg	171.07	0.91	M − H	HMDB0000721	Organic acids and derivatives	Carboxylic acids and derivatives	Amino acids, peptides, and analogues	−0.076 (−0.114–0.039)	<0.0001	0.005
neg_FT17207	3′-Sialyllactose	Neg	632.2	0.67	M − H	HMDB0000825	Organic oxygen compounds	Organooxygen compounds	Carbohydrates and carbohydrate conjugates	0.055 (0.026–0.084)	0.0003	0.012
pos_FT04847	Estrone sulfate	Pos	176.06	0.58	M + 2H	HMDB0001425	Lipids and lipid-like molecules	Steroids and steroid derivatives	Sulfated steroids	−0.056 (−0.087–0.025)	0.0004	0.014
pos_FT10083	Carnosine	Pos	249.09	0.49	M + Na	HMDB0000033	Organic acids and derivatives	Peptidomimetics	Hybrid peptides	−0.151 (−0.234–0.069)	0.0004	0.014
neg_FT01921	Formiminoglutamic acid	Neg	173.05	1.31	M − H	HMDB0000854	Organic acids and derivatives	Carboxylic acids and derivatives	Amino acids, peptides, and analogues	0.075 (0.032–0.118)	0.0007	0.020
pos_FT04858	Citrulline	Pos	176.1	0.78	M + H	HMDB0000904	Organic acids and derivatives	Carboxylic acids and derivatives	Amino acids, peptides, and analogues	0.048 (0.019–0.077)	0.001	0.028
neg_FT00788	4-Hydroxyproline	Neg	130.05	1.4	M − H	HMDB0000725	Organic acids and derivatives	Carboxylic acids and derivatives	Amino acids, peptides, and analogues	0.082 (0.031–0.132)	0.002	0.033
pos_FT09878	Hydroxyprolyl-Asparagine	Pos	246.1	0.69	M + H	HMDB0028858	Organic acids and derivatives	Carboxylic acids and derivatives	Amino acids, peptides, and analogues	−0.059 (−0.097–0.022)	0.002	0.033
pos_FT13592	2-Hexenoylcarnitine	Pos	296.12	6.85	M + K	HMDB0013161	Lipids and lipid-like molecules	Fatty Acyls	Fatty acid esters	0.104 (0.04–0.167)	0.002	0.033
pos_FT03157	L-Glutamine	Pos	147.07	0.52	M + H	HMDB0000641	Organic acids and derivatives	Carboxylic acids and derivatives	Amino acids, peptides, and analogues	−0.065 (−0.106–0.024)	0.002	0.033
neg_FT05602	Inosine	Neg	267.07	0.67	M − H	HMDB0000195	Nucleosides, nucleotides, and analogues	Purine nucleosides		0.038 (0.011–0.064)	0.005	0.073
neg_FT08407	N-(2-Hydroxyphenyl)acetamide glucuronide	Neg	328.06	3.81	M − H	HMDB0240542	Organic oxygen compounds	Organooxygen compounds	Carbohydrates and carbohydrate conjugates	0.081 (0.023–0.139)	0.006	0.092
pos_FT15625	Galactosylhydroxylysine	Pos	325.16	0.5	M + H	HMDB0000600	Organic acids and derivatives	Carboxylic acids and derivatives	Amino acids, peptides, and analogues	−0.048 (−0.084–0.013)	0.007	0.096

Model: metabolites = age + race + BMI z-score + total energy intake. Abbreviations: ID: metabolic feature identification; MZ, mass-charge; RT, retention time; HMDB, human metabolome database.

**Table 4:** List of significant metabolic features associated with positive change in BMI using the validation set feature in the replication set (1-year follow-up).

ID	Metabolites	Mode	MZ	RT	Adduct	HMDB ID	Super-Class	Class	Sub-Class	Replication Set		
										Estimate (95%CI)	*p*-Value	FDR adj
neg_FT01863	Glycylproline	Neg	173.09	0.75	M + H	HMDB0000721	Organic acids and derivatives	Carboxylic acids and derivatives	Amino acids, peptides, and analogues	−0.018 (−0.029–0.007)	0.002	0.031
neg_FT17207	3′-Sialyllactose	Neg	632.2	0.67	M − H	HMDB0000825	Organic oxygen compounds	Organooxygen compounds	Carbohydrates and carbohydrate conjugates	0.009 (0.002–0.016)	0.006	0.038
neg_FT01921	Formiminoglutamic acid	Neg	173.05	1.31	M − H	HMDB0000854	Organic acids and derivatives	Carboxylic acids and derivatives	Amino acids, peptides, and analogues	0.016 (0.004–0.028)	0.008	0.038
pos_FT04673	Glycylproline	Pos	171.07	0.91	M − H	HMDB0000721	Organic acids and derivatives	Carboxylic acids and derivatives	Amino acids, peptides, and analogues	−0.014 (−0.025–0.003)	0.01	0.038
neg_FT00788	4-Hydroxyproline	Neg	130.05	1.4	M − H	HMDB0000725	Organic acids and derivatives	Carboxylic acids and derivatives	Amino acids, peptides, and analogues	0.016 (0.003–0.03)	0.016	0.043
pos_FT04858	Citrulline	Pos	174.08	0.81	M − H	HMDB0000904	Organic acids and derivatives	Carboxylic acids and derivatives	Amino acids, peptides, and analogues	0.01 (0.002–0.018)	0.013	0.043
pos_FT09630	4-Vinylsyringol	Pos	243.09	0.73	M + H	HMDB0301746	Phenylpropanoids and polyketides	Stilbenes		−0.01 (−0.02–0.001)	0.022	0.049
neg_FT01967	Citrulline	Neg	176.1	0.78	M + H	HMDB0000904	Organic acids and derivatives	Carboxylic acids and derivatives	Amino acids, peptides, and analogues	0.012 (0.001–0.023)	0.025	0.049
neg_FT05602	Inosine	Neg	267.07	0.67	M − H	HMDB0000195	Nucleosides, nucleotides, and analogues	Purine nucleosides		0.005 (0.0004–0.01)	0.033	0.058
pos_FT10083	Carnosine	Pos	249.09	0.49	M + Na	HMDB0000033	Organic acids and derivatives	Peptidomimetics	Hybrid peptides	−0.015 (−0.031–0.0004)	0.056	0.082
neg_FT08407	N-(2-Hydroxyphenyl)acetamide glucuronide	Neg	328.06	3.81	M − H	HMDB0240542	Organic oxygen compounds	Organooxygen compounds	Carbohydrates and carbohydrate conjugates	0.015 (−0.0004–0.032)	0.056	0.082
pos_FT03157	L-Glutamine	Pos	147.07	0.52	M + H	HMDB0000641	Organic acids and derivatives	Carboxylic acids and derivatives	Amino acids, peptides, and analogues	−0.006 (−0.014–0.0001)	0.086	0.114
pos_FT04847	Estrone sulfate	Pos	176.06	0.58	M + 2H	HMDB0001425	Lipids and lipid-like molecules	Steroids and steroid derivatives	Sulfated steroids	−0.004 (−0.011–0.001)	0.139	0.171
pos_FT09878	Hydroxyprolyl-Asparagine	Pos	246.1	0.69	M + H	HMDB0028858	Organic acids and derivatives	Carboxylic acids and derivatives	Amino acids, peptides, and analogues	−0.001 (−0.009–0.006)	0.751	0.858
pos_FT15625	Galactosylhydroxylysine	Pos	325.16	0.5	M + H	HMDB0000600	Organic acids and derivatives	Carboxylic acids and derivatives	Amino acids, peptides, and analogues	−0.0008 (−0.008–0.007)	0.83	0.885
pos_FT13592	2-Hexenoylcarnitine	Pos	296.12	6.85	M + K	HMDB0013161	Lipids and lipid-like molecules	Fatty Acyls	Fatty acid esters	0.00 (−0.017–0.017)	1	1

Model: metabolites = age + race + BMI z-score + total energy intake. Abbreviations: ID, metabolic feature identification; MZ, mass-charge; RT, retention time; HMDB, human metabolome database.

## Data Availability

Data are contained within the article. The raw data supporting the conclusions of this article will be made available by the authors, upon reasonable request.

## Supplementary Materials

The following supporting information can be downloaded at: https://www.mdpi.com/article/10.3390/metabo13080899/s1, Figure S1: Principal component analysis (PCA) of 1532 annotated urinary metabolomics data with three groups (Discovery set (n = 235), Validation set (n = 125), and replication set (n = 81); Figure S2A: Principal Components analysis with outliers; Figure S2B: T^2^ Hotelling for I2 Principal Components; Table S1: Supplement Table S1. To study the association of metabolites with BMI z-score in discovery set and validation set; Supplement Table S2. Top enriched metabolic pathways using list of significant features in discovery set; Supplement Table S3. Top enriched metabolic pathways using list of significant features in the validation set; Supplement Table S4. Association of metabolic feature with BMI z-score in discovery set after adjusting for covariate (age, race, total energy intake); Supplement Table S5. Association of metabolic feature with BMI z-score in discovery set after adjusting for covariate (age, race, total energy intake, smoking, socioeconomic status)

## Abbreviations

BMI	Body mass index
UPLC–QTOF-MS	Ultra-high performance liquid chromatography-quadrupole time-of-flight mass spectrometry
FDR	False-discovery-rate
BTH	Buckeye Teen Health Study
OSU	Ohio State University
HPLC	High-performance liquid chromatography
ACN	Acetonitrile
4-NBA	4-nitrobenzoic acid
ESI	Electrospray ionization
QC	Quality control
CV	Coefficient of variation
MSTUS	Mass spectrometry total usable signal
HMDB	Human metabolome database
KEGG	Kyoto Encyclopedia of Genes and Genomes
CV	Coefficient of variation
MetPA	Metabolomic pathway enrichment analysis
AAs	Amino acids
BCAAs	Branched-chain amino acids
IGF-1	Insulin-like growth factor-1
cGP	cyclic Glycine-Proline
NEAAs	Non-essential amino acids
ROS	Reactive oxygen species
GSH	Glutathione
CVD	Cardiovascular diseases
